# Editorial: Critical- and high-priority pathogens in the food chain

**DOI:** 10.3389/fmicb.2025.1739491

**Published:** 2025-11-27

**Authors:** Daniel F. M. Monte, Fábio P. Sellera

**Affiliations:** 1 Department of Population Health and Pathobiology, North Carolina State University, College of Veterinary Medicine, Raleigh, NC, United States; 2 Department of Internal Medicine, School of Veterinary Medicine and Animal Science, University of São Paulo, São Paulo, Brazil; 3 School of Veterinary Medicine, Metropolitan University of Santos, Santos, Brazil

**Keywords:** food chain, antimicrobial resistance, horizontal gene transfer, plasmids, *Salmonella*, *Campylobacter*, *Listeria monocytogenes*, *Escherichia coli*

Foodborne bacterial pathogens pose a substantial threat to global public health by compromising food safety, economic stability, and the sustainability of production systems. Bacterial contamination can occur at various stages of the food chain, including primary production, processing, storage, and distribution, and can also be influenced by diverse environmental, biological, and management factors. Among these pathogens, antimicrobial-resistant isolates represent an additional and growing concern, as they can be acquired through the consumption of contaminated food, and infections caused by these agents reduce the effectiveness of available treatments. In this regard, the World Health Organization (WHO) Priority Pathogens List (2024) emphasizes critical and high-priority bacteria. However, the food chain harbors additional pathogens and factors that shape antimicrobial resistance (AMR) dynamics. This Research Topic compiles 13 articles that not only investigate priority pathogens but also expand the scope to include emerging microorganisms, socio-economic drivers, and innovative solutions for food safety. Together, these contributions provide a multidimensional perspective, highlighting both scientific advances and practical challenges.

## Priority foodborne pathogens and AMR

Krasteva et al. employed comparative proteomics to analyze *Listeria monocytogenes* strains of clinical and food origin, identifying proteins associated with virulence and host adaptation. The authors' findings contribute to our understanding of strain heterogeneity and may aid in the development of improved diagnostics or vaccine candidates.

Gahamanyi et al. characterized thermophilic *Campylobacter* spp. from humans, pigs, and chickens in Rwanda, revealing high levels of multidrug resistance, particularly in poultry isolates. These results underscore the urgent need for AMR surveillance in low- and middle-income countries, where poultry is both a major protein source and a potential reservoir for resistant bacteria.

Mohamed and Habib reviewed the distribution of virulence genes in *Salmonella* across Eastern and Southern Africa. In their work, the authors highlighted ecological and geographic variability, reinforcing the value of genomic surveillance and One Health approaches to guide tailored interventions in regions disproportionately affected by foodborne disease.

Fuga et al. investigated extended-spectrum β-lactamase (ESBL)-producing *Escherichia coli* in Brazilian retail meat, detecting ESBL-positive clones in both conventional and antibiotic-free production systems. The persistence of resistance even in antibiotic-free contexts demonstrates the complexity of AMR dissemination and the need for interventions that extend beyond farm-level antimicrobial use reduction.

Liu et al. examined an outbreak of enteroaggregative *E. coli* in Shandong Province, China, integrating epidemiological and genomic approaches. The authors identified strains co-harboring *mcr-1* and *bla*_CTX − M−132_, exemplifying the convergence of resistance to last-resort antimicrobials and underscoring the threat posed by extensively drug-resistant *E. coli* in human infections.

Tran et al. evaluated novel molecular markers to improve the detection of Shiga toxin-producing *E. coli* (STEC) in beef. Their proposed approach enhances the specificity of surveillance, ensuring better consumer protection while reducing unnecessary economic losses from false-positive results.

Ribeiro et al. investigated the conjugative transfer of *bla*_TEM_ from *Salmonella* Heidelberg to *E*. *coli* J53 AzR under conditions mimicking dairy and poultry processing environments. In a 3-h liquid-shaking protocol, the relative conjugation frequency (RCF) reached ~2.2% without supplements and increased by 3–4 orders of magnitude in chicken juice and whey, with whey-driven acidification associated with higher RCFs. The copper(II) complex Lu54 markedly reduced RCF (e.g., to ~0.3% without supplements; to ~1.7% in whey; and to ~0.9% in chicken juice); whole-genome sequencing of transconjugants suggested a loss of *bla*_TEM_-bearing plasmids after Lu54 exposure. These findings implicate food-processing byproducts as amplifiers of horizontal gene transfer while highlighting Lu54 as a promising mitigation strategy for milk and meat processing settings.

## Detection, surveillance, and molecular innovations

Zhang et al. developed a closed dumbbell-mediated isothermal amplification assay for the rapid detection of *L. monocytogenes*. The assay demonstrated high sensitivity and specificity, with potential for application in both laboratory and field settings. It represents a promising tool for routine food safety monitoring.

Complementing this, the molecular insights from Krasteva et al. and Liu et al. demonstrated how proteomic and genomic approaches not only elucidate pathogen biology but also strengthen outbreak investigation and AMR monitoring frameworks.

## Broader food safety and risk perspectives

Tang systematically reviewed microbial risks associated with spices, identifying contamination patterns in 41 common products. This study provides an evidence base for early warning systems, addressing a largely overlooked category of food that contributes significantly to international trade and dietary exposure.

Al Khatib and Kabir explored the role of socioeconomic status on the burden of foodborne diseases in the Middle East and North Africa. Their findings highlight how poverty and limited education exacerbate risks, particularly for parasitic infections, emphasizing the importance of social determinants in food safety policies.

Abdul and Pavoni conducted a bibliometric analysis of *Bacillus cereus* research in food safety. In their study, they mapped global trends and emerging themes such as cereulide toxicity and novel interventions, drawing attention to gaps in research on underexplored food matrices and the need for innovation in control strategies.

Li et al. described citrus black rot caused by *Alternaria alstroemeriae* and tested curcumin-loaded nanoliposomes as a postharvest control method. Their work demonstrates how natural compounds combined with nanotechnology can provide sustainable alternatives to synthetic fungicides for crop protection.

## Looking forward

Overall, this Research Topic illustrates the multifaceted challenges of AMR and foodborne pathogens along the food chain. The contributions span priority bacteria, emerging risks, and enabling technologies, while considering broader social and economic drivers. Moving forward, three priorities emerge: (i) strengthening integrated, One Health–based surveillance that links animals, food, environments, and humans; (ii) pairing molecular epidemiology with risk assessment to guide proportionate interventions from farm to fork; and (iii) investing in feasible, scalable mitigation strategies, including hygienic design, process controls, and consumer-level education, tailored to diverse resource settings.

